# Oxaloacetate treatment preserves motor function in SOD1^G93A^ mice and normalizes select neuroinflammation-related parameters in the spinal cord

**DOI:** 10.1038/s41598-021-90438-6

**Published:** 2021-05-26

**Authors:** Sudheer K. Tungtur, Heather M. Wilkins, Robert S. Rogers, Yomna Badawi, Jessica M. Sage, Abdulbaki Agbas, Omar Jawdat, Richard J. Barohn, Russell H. Swerdlow, Hiroshi Nishimune

**Affiliations:** 1 Department of Anatomy and Cell Biology, School of Medicine, University of Kansas, Kansas City, KS 66160 USA; 2 Department of Neurology, School of Medicine, University of Kansas, Kansas City, KS 66160 USA; 3 Department of Basic Sciences, Kansas City University, Kansas City, MO 64106 USA; 4 Present Address: Cardiovascular Division, University of Minnesota School of Medicine, Minneapolis, MN 55455 USA; 5 Present Address: Department of Curriculum and Integrative Learning, Kansas City University, Joplin, MO 64804 USA; 6 Present Address: Department of Neuroscience, University of Pittsburgh, Pittsburgh, PA 15260 USA; 7 Present Address: Department of Neurology, University Missouri-Columbia, Columbia, MO 65212 USA; 8 Present Address: Tokyo Metropolitan Institute of Gerontology, Neurobiology of Aging, 35-2 Sakaecho, Itabashi-ku, Tokyo, 173-0015 Japan

**Keywords:** Amyotrophic lateral sclerosis, Amyotrophic lateral sclerosis

## Abstract

Amyotrophic lateral sclerosis (ALS) remains a devastating motor neuron disease with limited treatment options. Oxaloacetate treatment has a neuroprotective effect in rodent models of seizure and neurodegeneration. Therefore, we treated the ALS model superoxide dismutase 1 (SOD1) ^G93A^ mice with oxaloacetate and evaluated their neuromuscular function and lifespan. Treatment with oxaloacetate beginning in the presymptomatic stage significantly improved neuromuscular strength measured during the symptomatic stage in the injected mice compared to the non-treated group. Oxaloacetate treatment starting in the symptomatic stage significantly delayed limb paralysis compared with the non-treated group. For lifespan analysis, oxaloacetate treatment did not show a statistically significant positive effect, but the treatment did not shorten the lifespan. Mechanistically, SOD1^G93A^ mice showed increased levels of tumor necrosis factor-α (TNFα) and peroxisome proliferative activated receptor gamma coactivator 1α (PGC-1α) mRNAs in the spinal cord. However, oxaloacetate treatment reverted these abnormal levels to that of wild-type mice. Similarly, the altered expression level of total NF-κB protein returned to that of wild-type mice with oxaloacetate treatment. These results suggest that the beneficial effects of oxaloacetate treatment in SOD1^G93A^ mice may reflect the effects on neuroinflammation or bioenergetic stress.

## Introduction

Amyotrophic lateral sclerosis (ALS) is characterized by progressive motor function weakness from upper and lower motor neuron degeneration^1–3^. One of the most studied familial ALS types is caused by mutation of the superoxide dismutase 1 (SOD1) gene on chromosome 21. The SOD1 mutation reportedly accounts for approximately 20% of all familial ALS cases. SOD1 is classically considered a cytoplasmic enzyme, although recent reports show localization of SOD1 within mitochondrial membranes for both mutant SOD1 and, to a lesser extent, wild-type SOD1^4^. The ALS mouse model SOD1^G93A^ transgenic mice have altered mitochondrial morphology and mitochondrial SOD1 accumulation^5,6^. These phenotypes raise the possibility that mutant SOD1 may drive neurodegeneration by damaging mitochondria. Mitochondrial dysfunction is functionally relevant for ALS neurodegeneration^7,8^. SOD1^G93A^ mice show mitochondrial perturbations, including vacuolization^9^ and mitochondrial protein transport defects^10^. The mitochondrial ultrastructure is perturbed in ALS^11,12^. Cytoplasmic inclusions (Bunina bodies) that may represent mitochondria-containing autophagic vacuoles are observed in ALS motor neurons^13–16^. Electron transport chain activities are reduced, and mitochondrial DNA is altered in the ALS spinal cord^17^. A reduction in mitochondrial complex I activity and mitochondrial DNA perturbation were corroborated in studies of muscles in sporadic ALS patients^18,19^. These data suggest a role for mitochondrial dysfunction in ALS etiology, as reviewed in many recent publications^1,11,20–22^.

Oxaloacetate plays a role in cell bioenergetics, and its administration affects bioenergetics-relevant infrastructure. Systemic administration of oxaloacetate to wild-type mice increased the brain expression level of PGC1α, a transcriptional co-activator and a master-coordinator of mitochondrial biogenesis^23^. Furthermore, oxaloacetate altered the levels, distributions, or post-translational modifications of mRNA and proteins in ways that promote mitochondrial biogenesis. This oxaloacetate effect has been observed through effects on PGC1 related co-activator, nuclear respiratory factor 1, transcription factor A of the mitochondria, cytochrome oxidase subunit 4 isoform 1, cAMP-response element binding protein, p38 MAPK, and AMP-activated protein kinase^23^. Oxaloacetate can penetrate the blood–brain barrier and access the central nervous system following systemic administration^23,24^. Furthermore, it shows neuroprotective effects in a neurodegenerative mouse model generated by kainic acid injection^24^ and prolongs *C. elegans* survival by mimicking caloric restriction^25^. Neuroprotective effects have also been observed with a combined treatment of recombinant Glu-oxaloacetate-transaminase and its co-factor oxaloacetate in a neurodegenerative disease rat model generated by kainic acid/cyclothiazide injection^26^. However, oxaloacetate has not been tested alone in transgenic rodent models of ALS. In this study, we treated SOD1^G93A^ mice with oxaloacetate starting from the presymptomatic stage or the symptomatic stage and analyzed both neuromuscular function and lifespan. We also analyzed gene expression levels in spinal cords and mitochondrial respiratory function to reveal the mechanisms that could mediate the effects of oxaloacetate treatment on SOD1^G93A^ mice.

## Results

### Oxaloacetate treatment improves neuromuscular strength

The effect of oxaloacetate was tested in SOD1^G93A^ mice that received intraperitoneal injection of two grams oxaloacetate per kg body weight, once per day, five days a week, from the two starting ages described below till the end-stage. This dosing regimen was based on our previous oxaloacetate injection project in wild-type mice^23^. For SOD1^G93A^ mice with the C57Bl/6 J background, an early stage with some motor performance deficits occurs between 50 and 60 days of age^27,28^, and disease onset occurs between 91 and 111 days of age as indicated by the emergence of limb tremor^28,29^. The survival rate starts to fall below 100% at approximately 125–130 days of age^27,30^, and the mean survival ages reported in four previous papers ranged between 142 and 161 days^27–30^. Based on this phenotypic timeline, the effect of oxaloacetate in SOD1^G93A^ mice was compared in three groups with 12 mice per group: a group treated from the presymptomatic stage at 60 days of age, a group treated from the symptomatic stage at 110 days of age^2^, and a control group without treatment. Oxaloacetate was administered by intraperitoneal injection, which may elicit minor pain and immunological reaction in the peritoneal cavity resulting in increased stress for the animal. Therefore, we chose to use non-treated SOD1^G93A^ mice as controls to evaluate the overall effect of oxaloacetate administration.

**Figure 1:**
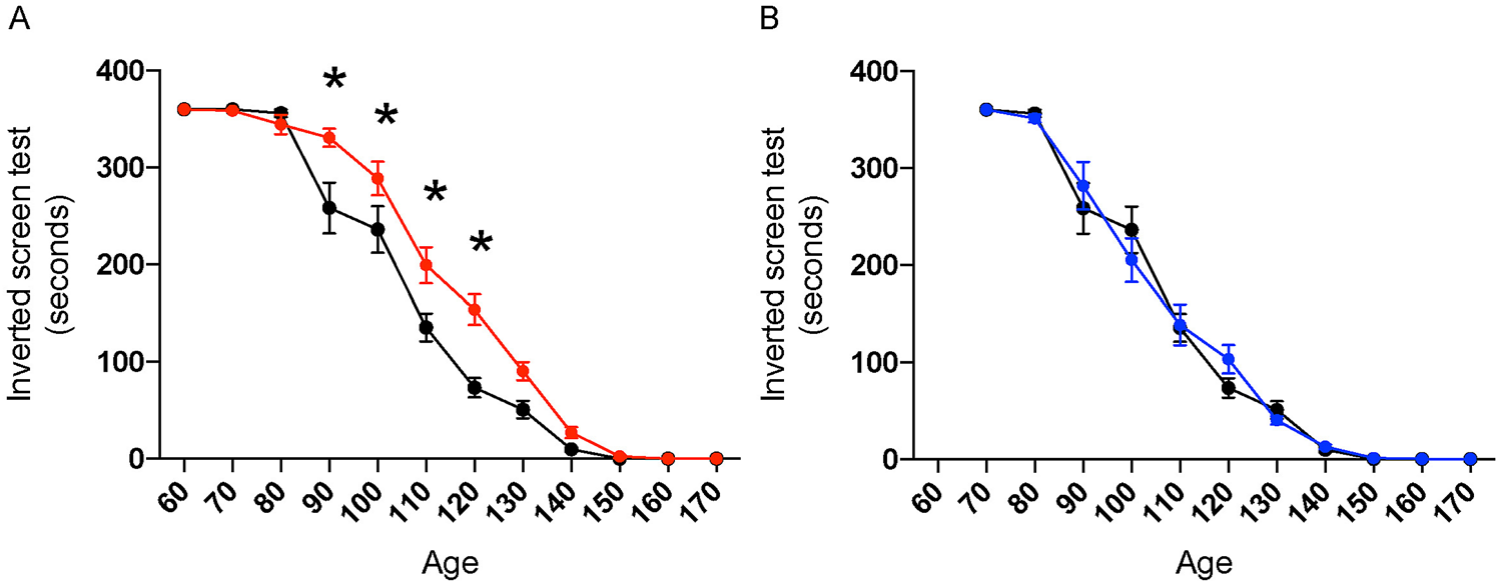
Oxaloacetate treatment from the presymptomatic stage improves the neuromuscular strength of SOD1^G93A^ mice in the symptomatic stage. (**A**) Injection of oxaloacetate at 2 g/kg body weight/5 days a week from the presymptomatic stage (60 days old, red) delayed the decline in neuromuscular function measured using an inverted screen test. The vertical axis shows the total hanging time from three trials of the inverted screen test, and the horizontal axis indicates the age of measurement. The line graphs show mean ± S.E.M, which were for oxaloacetate-injected group 330.8 ± 9.3 versus control group 258.4 ± 26.2 at age 90, 288.8 ± 17.4 versus 236.1 ± 24 at age 100, 199.4 ± 18.5 versus 135.5 ± 14.5 at age 110, 153.8 ± 15.7 versus 73.58 ± 10.1 at age 120. Asterisks indicate a significant difference between the oxaloacetate injected (red) and control group of non-injected (black) SOD1^G93A^ mice by repeated measure two way-ANOVA, *p* < 0.01. (**B**) The group treated from the symptomatic stage (110 days old, blue) did not show differences from the control group of non-injected SOD1^G93A^ mice (black, same mice as **A**). *N* = 12 mice per group.

Neuromuscular strength was measured using an inverted screen test performed every ten days, beginning at 60 days of age. The group treated from the presymptomatic stage showed significantly preserved neuromuscular function compared to the control group between 90 and 120 days of age, as revealed by a longer duration of hanging in the inverted body position (Fig. 1A). The inverted screen test hanging time improved by 28.0% at 90 days of age, 22.3% at 100 days of age, 47.2% at 110 days of age, and 109.0% at 120 days of age. At 130 to 140 days of age, the oxaloacetate-injected group showed a longer mean hanging time compared to the non-injected group, but the difference was not statistically significant. At age 150- to 160 days, there were no differences between the groups injected in the presymptomatic stage and the non-injected group. The group that started oxaloacetate treatment at the 110-day-old symptomatic stage did not show a beneficial effect between 110 and 160 days of age on the inverted screen test (Fig. 1B).

### Oxaloacetate treatment delays the onset of neurological symptoms.

A healthy wild-type mouse will stretch out its hind limbs in the air when it is hung by the tail. If a mouse has neuromuscular degeneration in its hind limbs, the hind limbs fold close to the body when the mouse is hung by the tail, and this phenotype is given a neurological score of 1 in ALS preclinical studies^31–33^. In this study, median ages for a neurological score of 1 were 81.5 days for the presymptomatic stage treatment group, 90 days for the symptomatic stage treatment group, and 81 days for the non-injected controls. However, the differences were not statistically significant, suggesting that the oxaloacetate treatment did not affect the onset of a neurological score of 1 by the log-rank test. In addition, the median age of 90 for the symptomatic stage treatment group indicated that the oxaloacetate treatment started post-symptom onset for this group.

**Figure 2:**
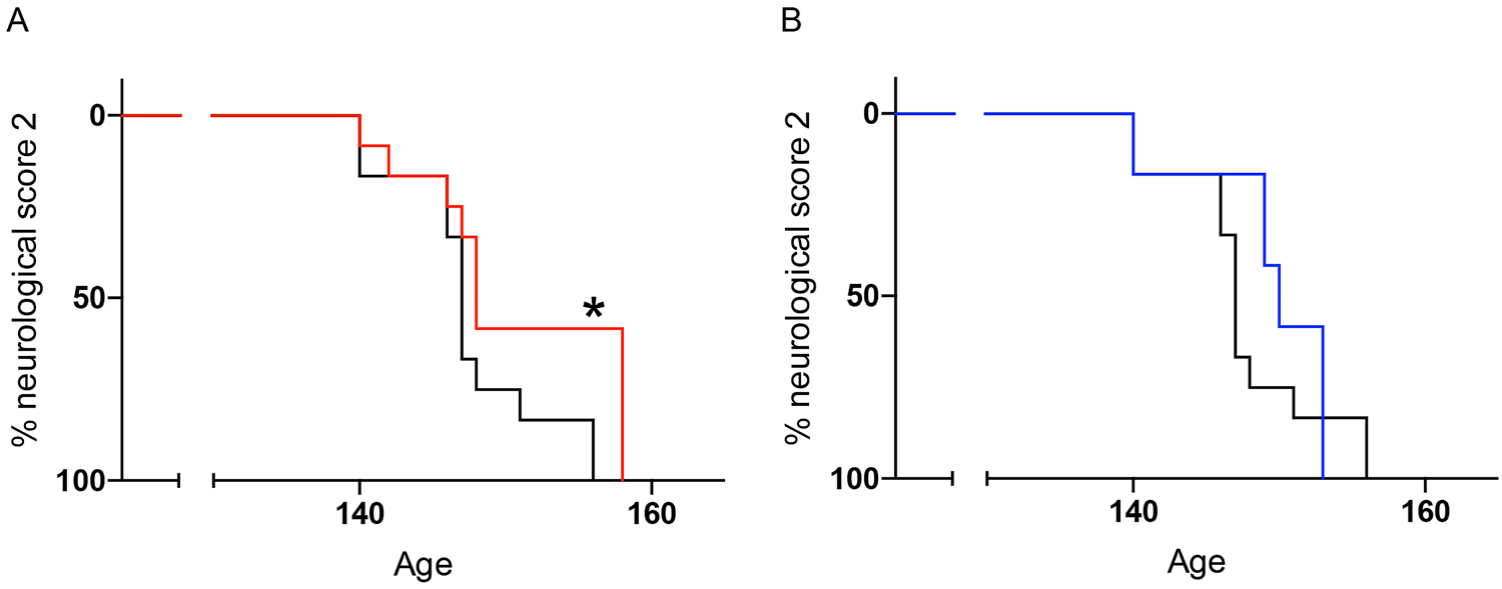
Oxaloacetate treatment from the presymptomatic stage delays the toe-curling phenotype (neurological score of 2) of SOD1^G93A^ mice. (**A**) Oxaloacetate treatment from the presymptomatic stage (60 days old, red) delayed the median of the onset age of a neurological score of 2 by one day compared to the control group of non-injected SOD1^G93A^ mice (black, *p* = 0.0448, log-rank test). (**B**) Oxaloacetate treatment from the symptomatic stage (110 days old, blue) did not alter the median onset age of a neurological score of 2. (*p* = 0.3420, log-rank test). The graphs show Kaplan–Meier curves with *n* = 12 mice per group. These graphs contain mice that are identical to the mice in Fig. 1.

The onset of the toe-curling phenotype (neurological score of 2) was significantly different by log-rank test (*p* = 0.0448, Fig. 2A) between the presymptomatic stage treatment group (median age 148 days) and the control group (median age 147 days), which was a 0.7% increase. The onset of a neurological score of 2 for the symptomatic stage treatment group was not significantly different compared to the controls (*p* = 0.1383, median age 150, Fig. 2B).

**Figure 3:**
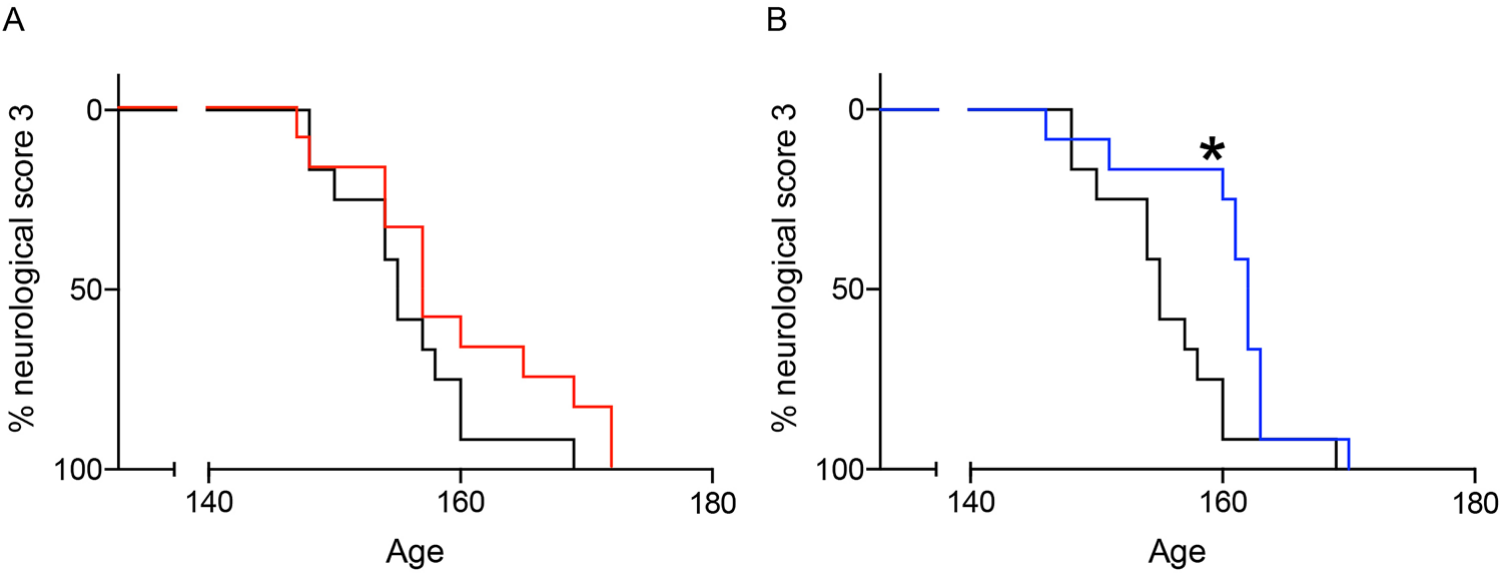
Oxaloacetate treatment from the symptomatic stage delays the onset of limb paralysis (neurological score of 3) of SOD1^G93A^ mice. (**A**) Oxaloacetate treatment from the presymptomatic stage (60 days old, red) did not alter the median age of onset of limb paralysis of SOD1^G93A^ mice compared to the control group of non-injected SOD1^G93A^ mice (black, *p* = 0.1793, log-rank test). (**B**) Oxaloacetate treatment from the symptomatic stage (110 days old, blue) significantly delayed the median age of onset for limb paralysis by seven days (*p* = 0.0157, log-rank test). The graphs show the Kaplan–Meier curve with *n* = 12 mice per group. These graphs contain mice that are identical to the mice in Fig. 1.

Importantly, the onset of limb paralysis (neurological score of 3) was delayed significantly by seven days in the symptomatic stage treatment group. The median age of onset of a neurological score of 3 was 162 days for the symptomatic stage treatment group and 155 days for the non-treated control group), which was a 4.5% increase (*p* = 0.0157, log-rank test). The median age of onset of a neurological score of 3 was 157 days for the presymptomatic stage treatment group, which was not significantly different from the controls (Fig. 3).

**Figure 4:**
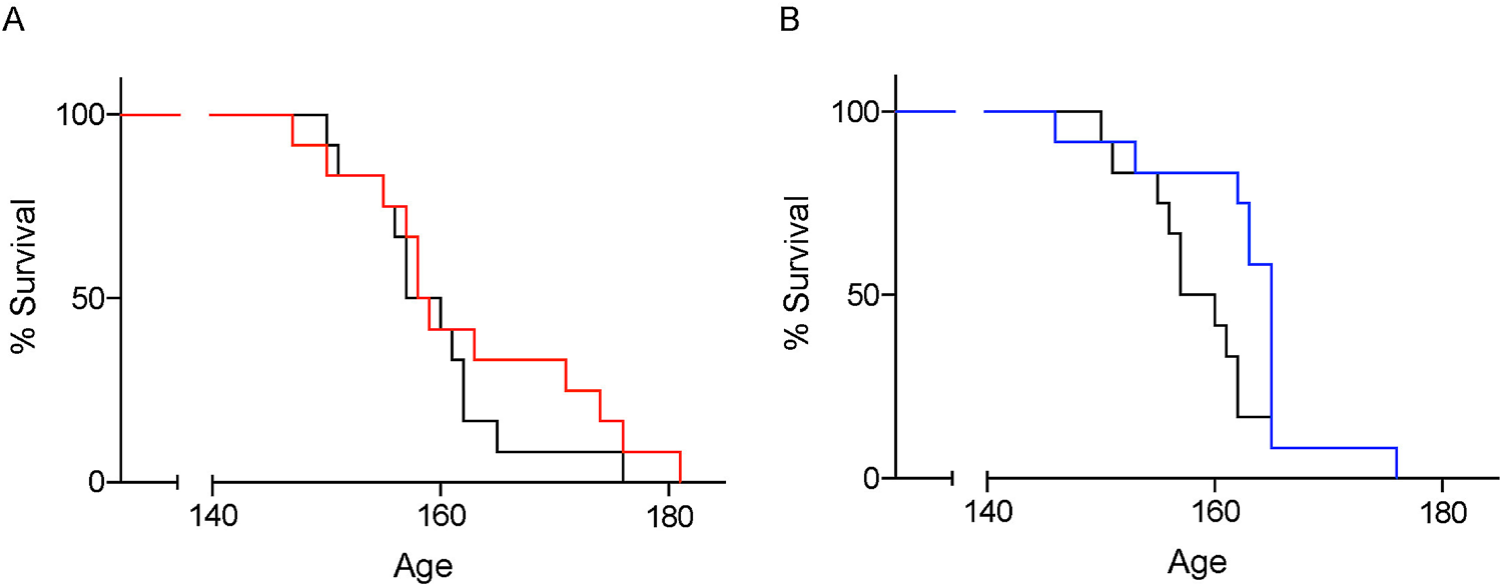
The lifespan of oxaloacetate-treated SOD1^G93A^ mice. (**A**) The median end-stage age of oxaloacetate treatment of SOD1^G93A^ mice from the presymptomatic stage (60 days old, red) was 158.5, which was the same as the control group of non-injected SOD1^G93A^ mice (black, *p* = 0.3717, log-rank test). (**B**) The median end-stage age of oxaloacetate treatment from the symptomatic stage (110 days old, blue) was 165 days. This median age was not statistically different from the control group (*p* = 0.051, log-rank test). The graphs show Kaplan–Meier curves with *n* = 12 mice per group. These graphs contain mice that are identical to the mice in Fig. 1.

For lifespan analysis, the end-stage was defined using the righting reflex. The median lifespan age of the symptomatic stage treatment group was 165 days, and the non-injected control group was 158.5 days (Fig. 4B). However, this was not a statistically significant effect (*p* = 0.0510 by log-rank test). In the presymptomatic stage treatment group, the median lifespan age was 158.5 days and was the same as controls (Fig. 4A). Neither the presymptomatic nor symptomatic treatment groups showed a negative effect on SOD1^G93A^ mouse lifespan as compared to the non-injected group at the dose used in this study.

### Mitochondrial respiratory function in oxaloacetate-treated mice

**Figure 5:**
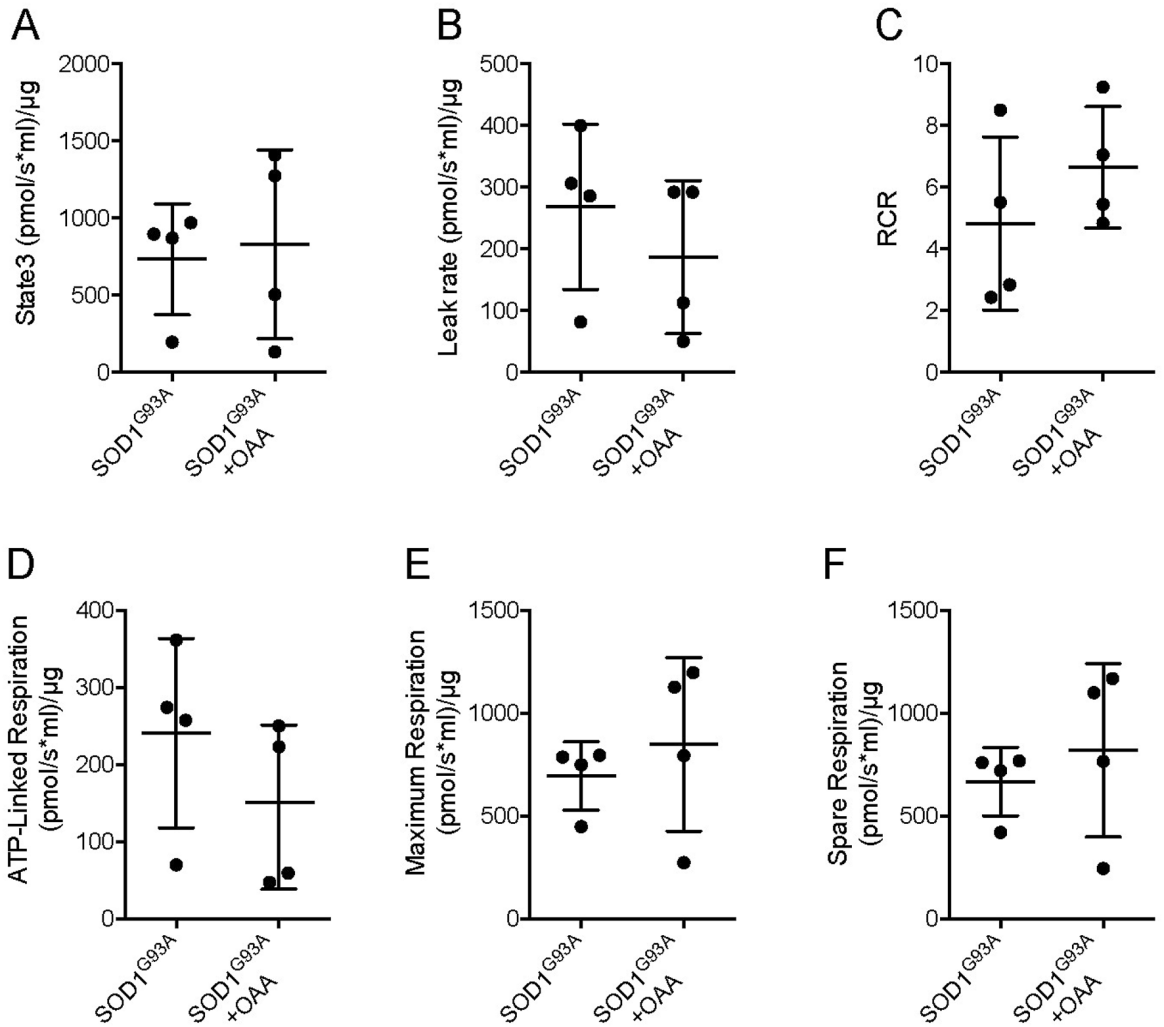
Mitochondrial respiratory function in brain homogenates of SOD1^G93A^ mice with or without oxaloacetate (OAA) treatment. (**A**) The respiration rate obtained after addition of ADP. (**B**) The leak respiration rate after oligomycin injection. (**C**) Respiratory control ratio (RCR) obtained by dividing the state 3 by the leak rate. Respiration rate for (**D**) ATP-linked component, (**E**) maximum component, and (**F**) spare component. The graphs show data from four mice with whiskers showing mean ± S.D. There were no statistically significant differences with or without oxaloacetate treatment (*n* = 4, mean ± S.D. and unpaired t test *p values* were (**A**) SOD1^G93A^, 732.9 ± 360.1, SOD1^G93A^ + OAA, 829.4 ± 611.6, *p* = 0.7947; (**B**) SOD1^G93A^, 268.2 ± 133.8, SOD1^G93A^ + OAA, 186.6 ± 124, *p* = 0.4056; (**C**) SOD1^G93A^, 4.8 ± 2.8, SOD1^G93A^ + OAA, 6.6 ± 2.0, *p* = 0.3282; (**D**) SOD1^G93A^, 240.9 ± 122.7, SOD1^G93A^ + OAA, 145.1 ± 106.4, *p* = 0.2825; (**E**) SOD1^G93A^, 695.3 ± 165.6, SOD1^G93A^ + OAA, 848 ± 421.2, *p* = 0.5251; and (F) SOD1^G93A^, 667.1 ± 165.6, SOD1^G93A^ + OAA, 819.8 ± 421.2, *p* = 0.5251).

Next, we considered mechanisms that might mediate the beneficial effects of oxaloacetate treatment. For these mechanistic analyses, oxaloacetate was administered to SOD1^G93A^ mice by intraperitoneal injection from the presymptomatic stage at 60 days of age with the same dose and frequency. The mice were dissected at 130 days of age to analyze the tissue during the symptomatic stage before the end-stage. Our previous publication showed that intraperitoneal injection of oxaloacetate in wild-type mice enhanced mitochondrial biogenesis signaling pathways in the brain^23^. In the current study, we investigated the effect of oxaloacetate on mitochondrial respiratory function in the brains of SOD1^G93A^ mice using an Oroboros respirometer. Respiratory parameters were not significantly altered by oxaloacetate treatment, measured in the brains of four mice per group (Fig. 5).

### Oxaloacetate treatment reduces neuroinflammation in the spinal cord

**Figure 6:**
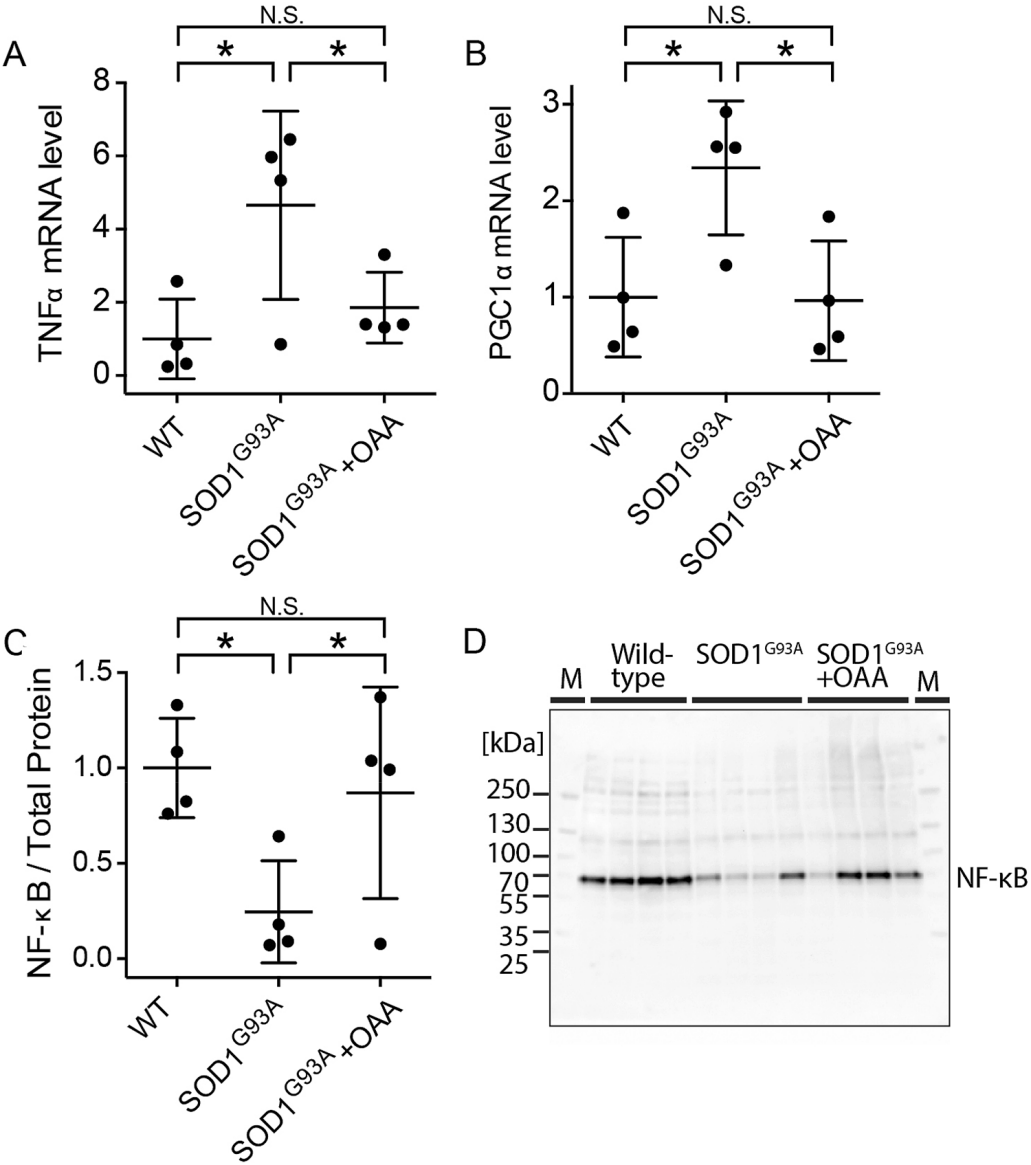
Oxaloacetate (OAA) treatment reverts TNFα, PGC-1α, and NF-κB expression levels in SOD1^G93A^ mice to wild-type mice levels. Expression levels of (**A**) TNFα mRNA (*Tnf*) and (**B**) PGC-1α mRNA (*Ppargc1a*) in the thoracic spinal cords were analyzed using TaqMan qPCR and normalized to actin mRNA level. *Tnf* and *Ppargc1a* expression levels showed increases in SOD1^G93A^ mice, which reverted to wild-type mice levels following oxaloacetate treatment. (**C**) NF-κB protein expression levels in lumbar spinal cords of the same animals as (**A** and **B**) were analyzed by western blot as shown in (**D**). NF-κB expression levels showed a decreased level in SOD1^G93A^, which reverted to the wild-type level following oxaloacetate treatment. An image of one full-length blot is shown in (**D**). Molecular weight markers were run in lanes marked “M,” and the molecular weights are indicated on the left side of the blot. The graphs show data from four mice with whiskers showing mean ± S.D. for WT, SOD1^G93A^, SOD1^G93A^ + OAA: (A, 1.0 ± 1.1, 4.7 ± 2.6, 1.9 ± 1.0; B, 1.0 ± 0.6, 2.3 ± 0.7, 1.0 ± 0.6; C, 1.0 ± 0.3, 0.2 ± 0.3, 0.9 ± 0.6). The differences among the means are statistically significant (*) by one-way ANOVA (6A, *p* = 0.0343; 6B, *p* = 0.0229; 6C, *p* = 0.0475) with Fisher’s LSD multiple comparisons (*n* = 4, *p* < 0.05). N.S.: not significant.

To investigate other potential oxaloacetate-induced molecular changes, we compared the levels of cytokines and signaling pathways related to neuroinflammation in SOD1^G93A^ mice, SOD1^G93A^ mice treated with oxaloacetate, and wild-type C57Bl/6 J mice with matching age and sex (Fig. 6A). The SOD1^G93A^ mice and SOD1^G93A^ mice treated with oxaloacetate were the same animals used in Fig. 5. Entire brains were necessary for the mitochondrial respiratory analysis; therefore, spinal cords were used for this analysis. Neuroinflammatory cytokine tumor necrosis factor-α (TNFα) mRNA (*Tnf*) levels were increased in SOD1^G93A^ mice compared to those in the wild-type mice. However, oxaloacetate treatment restored the elevated *Tnf* level in SOD1^G93A^ mice to that of the wild-type mice.

Peroxisome proliferator activated receptor gamma coactivator 1α (PGC-1α) mRNA (*Ppargc1a*) expression levels changed in the same manner as *Tnf* mRNA. *Ppargc1a* levels were increased in SOD1^G93A^ mice compared to those in the wild-type mice, but oxaloacetate treatment reduced *Ppargc1a* mRNA to wild-type mice levels (Fig. 6B). The PGC-1α protein levels, measured by western blot analysis, were lower in oxaloacetate-treated SOD1^G93A^ mice compared to untreated SOD1^G93A^ mice in three out of four mice. Still, the average levels of four mice per group were not statistically different (*n* = 4, unpaired t-test *p* = 0.1380). Interestingly, the nuclear factor kappa-light-chain-enhancer of activated B (NF-κB) protein level was lower in spinal cords of SOD1^G93A^ mice than in wild-type mice. Oxaloacetate treatment increased the total NF-κB protein levels of SOD1^G93A^ mice to the wild-type mouse level (Fig. 6C, D). These results demonstrate that oxaloacetate treatment returned abnormal levels of neuroinflammatory cytokines and signaling pathways related to inflammation in the spinal cords of SOD1^G93A^ mice to levels measured in wild-type mice.

## Discussion

In this study, SOD1^G93A^ mice were treated with oxaloacetate from the presymptomatic stage (60 days old) or the symptomatic stage (110 days old). For some measures, treatment initiated during the presymptomatic stage maintained neuromuscular strength and delayed the toe-curling phenotype appearance. Treatment initiated during the symptomatic stage delayed the onset of limb paralysis. These results suggest that oxaloacetate treatment improves neuromuscular function during the symptomatic stages and significantly delays functional decline. Neither treatment paradigm significantly extended lifespan or accelerated disease symptoms. An analysis of temporally concordant molecular changes revealed that oxaloacetate treatment reversed abnormal levels of TNFα mRNA, PGC-1α mRNA, and NF-κB protein in the spinal cords of SOD1^G93A^ mice to wild-type mice levels. These results suggest that oxaloacetate’s beneficial effect on the motor function in SOD1^G93A^ mice may reflect effects on neuroinflammation or effects on bioenergetic stress. When considered from the perspective that SOD1^G93A^ mice experience various molecular stresses and oxaloacetate tended to benefit SOD1^G93A^ motor function, it seems likely that oxaloacetate treatment minimized some stress parameters.

The biological effect of oxaloacetate has been previously tested in several animal models, and beneficial effects have been reported. Oxaloacetate has been shown to penetrate the blood–brain barrier and access the central nervous system during systemic administration to wild-type mice^23,24^. Oxaloacetate treatment in wild-type mice has altered levels, distribution patterns, or post-translational modifications of mRNA and proteins in ways that promote mitochondrial biogenesis, including PGC1α, a transcriptional co-activator, and a master-coordinator of mitochondrial biogenesis^23^. In a mouse model of seizures induced by kainic acid injection, oxaloacetate helps ameliorate brain mitochondrial DNA damage and seizures. Oxaloacetate has also been proposed to inhibit reactive oxygen species-dependent oxidative damage in cells^24^. Similarly, in a neurodegenerative rat model generated by injecting kainic acid and cyclothiazide, combined treatment of oxaloacetate and recombinant Glu-oxaloacetate-transaminase showed protection of spinal cord motor neurons, a delayed weakening of motor functions, and prolonged survival^26^. As a potential mechanism, the authors proposed that co-treatment of Glu-oxaloacetate transaminase and its co-factor oxaloacetate increases glutamate scavenger activity in the bloodstream, which increases efflux of glutamate from the spinal cord to the blood and mitigates the deleterious effects of glutamate in the spinal cord. In a *C. elegans* aging model, oxaloacetate treatment extended the lifespan^25^. It appeared to function as a caloric restriction mimetic through AMP kinase (AMPK) and forkhead O-box (FOXO) transcription factor-dependent pathways. These results suggest that oxaloacetate treatment may exert a beneficial effect through multiple pathways, as discussed previously^23^.

In the current study, respiratory parameters were similar between SOD1^G93A^ mice with or without oxaloacetate treatment. However, oxaloacetate treatment decreased the PGC1α mRNA level in SOD1^G93A^ mice to that of wild-type mice. Therefore, oxaloacetate does not appear to have activated mitochondrial biogenesis in SOD1^G93A^ mice. The treatment-associated decrease in PGC1α mRNA levels may suggest that oxaloacetate minimizes mitochondrial biogenesis or bioenergetic stress in SOD1^G93A^ mice.

Importantly, oxaloacetate treatment could revert the elevated TNFα mRNA levels in the spinal cord of SOD1^G93A^ mice down to that of the wild-type mice (Fig. 6). We could not analyze the protein levels of TNFα due to a lack of a reliable antibody for western blot analysis. Downregulation of TNFα levels in the spinal cord of SOD1^G93A^ mice may reflect reduced inflammation or actively reduce inflammation. In this case, the therapeutic benefits we observed are pertinent to a recent RNA-seq analysis that identified TNF-mediated inflammation as a major abnormality in human ALS patients’ spinal cords^34^. Brohawn et al. proposed reducing inflammatory TNFα signaling might help ALS patients. Our preclinical data suggest oxaloacetate treatment is a promising intervention approach for ALS, although further studies are required before a clinical trial. Caution is certainly needed on this point, as TNFα knockout mice crossed with SOD1^G93A^ mice did not show improved motor neuron survival or lifespan^35^. This null mutation approach may complicate the outcome because a lack of TNFα protein from the developmental stage may induce compensation by other ligands of TNFR1 and TNFR2, including LTβ^36^.

In addition, oxaloacetate treatment returned the elevated PGC-1α mRNA level in the spinal cord of SOD1^G93A^ mice down to that of the wild-type mice (Fig. 6). Overexpression of PGC-1α in microglia and neuroblastoma cells is known to suppress the expression of neuroinflammatory cytokines^37,38^. Increased spinal cord *Ppargc1a* expression in SOD1^G93A^ mice may reflect an attempt to decrease TNFα due to the increased expression level of TNFα. This interpretation is consistent with our data that show *Tnf* and *Ppargc1a* expression reverts to wild-type levels following oxaloacetate treatment.

Finally, oxaloacetate treatment increased the total NF-κB protein level in the spinal cord of SOD1^G93A^ mice to the level seen in the wild-type mice (Fig. 6). Others have also published western blot analyses of total NF-κB protein levels in the lumbar spinal cords of SOD1^G93A^ mice, which decreases the levels from the presymptomatic stage to the symptomatic stage (Fig. 1 in reference^39^). Restoration of total NF-κB protein levels in the spinal cord lysates of oxaloacetate-treated SOD1^G93A^ mice may be beneficial for the neuroinflammation in the spinal cord. This view is consistent with the publication by Frakes et. al. who showed a beneficial effect of decreasing NF-κB signaling in microglia on motor neuron survival and survival of SOD1^G93A^ mice^39^. However, the level of activated NF-κB needs to be investigated to draw further conclusions of oxaloacetate treatment. These analyses of signaling molecules suggest that the potential modulation of TNFα and NF-κB signaling pathways either (a) mediates the beneficial effects or (b) acts as a biomarker for other beneficial mechanisms of oxaloacetate treatment in SOD1^G93A^ mice.

In conclusion, oxaloacetate treatment maintained neuromuscular function and delayed neurological symptom onset in SOD1^G93A^ mice when treated from the presymptomatic stage and delayed limb paralysis when treated from the symptomatic stage. At the dose tested, oxaloacetate treatment neither shorten nor extend the lifespan of SOD1^G93A^ mice to a statistically significant extent. Thus, neuromuscular function improvements of SOD1^G93A^ mice following oxaloacetate treatment did not come at the expense of a shortened lifespan. The limitation of the current study is that some of the beneficial effects were observed when the oxaloacetate treatment was initiated in the presymptomatic stage, which is difficult to achieve for most ALS patients. However, when the treatment started in the symptomatic stage, the oxaloacetate treatment delayed the limb paralysis onset. This beneficial effect of oxaloacetate treatment may delay the loss of patients’ neuromuscular function and movement ability. Such beneficial effects have a potentially significant impact on the quality of life for patients.

## Methods

### Animals

Animal experimental protocols were approved by the Institutional Animal Care and Use Committee of the University of Kansas Medical Centre (KUMC). All experiments were performed in accordance with the Guidelines for the Care and Use of Laboratory Animals of KUMC and the approved animal care and use protocol. The authors complied with the ARRIVE 2.0 guidelines. Male SOD1^G93A^ mice with a C57Bl/6 J background (B6.Cg-Tg (SOD1*G93A) 1 Gur/J, the Jackson Laboratory stock number 004435, high copy number transgene)^5,40^ were obtained from the Jackson Laboratory and used in this study without breeding. The Jackson Laboratory confirms the transgene copy number using quantitative PCR (qPCR, the Jackson Laboratory technical information). There is no difference in survival between males and females in the B6 background^30^. The incidence of human ALS is higher in males^41,42^; thus, only male mice were analyzed in this study. The Guidelines for ALS preclinical animal research published based on the consensus meeting recommends using 12 or more animals of single-sex for survival analysis^32^. Thus, twelve mice per group and a total of 36 singly housed male mice were analyzed. Purchased mice were randomized into three groups for the analysis. Controls were genotype-, age- and sex-matched mice. The end-stage of a lifespan was recorded when mice are unable to right themselves 30 s after having been pushed on their side based on the guidelines for preclinical studies using ALS mouse models^31,32^.

Oxaloacetic acid (Sigma-Aldrich, #O7753) was reconstituted on the day of injection in PBS, adjusted to a final pH of 7.2 – 7.4 with sodium hydroxide, filter sterilized, and injected into the mice as soon as possible. Oxaloacetate was administered by intraperitoneal injection, 2 g/kg body weight, with one injection per day for five days a week. The drug dose, injection method, and frequency are based on the successful outcomes of oxaloacetate administration in wild-type mice in our published study^23^.

### Neurological score

Disease onset was estimated based on the age of peak body weight, which was evaluated every ten days. Disease progression and end-stage were assessed using the neurological scoring system described by Leitner et al.^31^ with the following criteria: (0) hind limb stretch similar to wildtype mice when suspended by its tail, (1) tail hang + leg spray test in which the tail hang caused a collapse, partial collapse or trembling of the hind limbs, (2) toes curl under at least twice during walking for 12 inches, (3) rigid paralysis of the hind limbs with the foot not being used for forwarding motion, and (4) the mouse cannot right itself within 30 s from either side. For survival assays, the end-stage was defined as the age at which mice showed the neurological score (4). The neurological score evaluation was performed at the time of daily injection of oxaloacetate by the person performing the injection.

### Inverted screen test

Evaluation of muscle strength: For SOD1^G93A^ mice, muscle strength was evaluated using an inverted screen test ^43,44^. In brief, the mouse was placed in the center of stainless-steel wire mesh. Then, the screen was smoothly inverted and held ∼40 cm above a soft surface. The time until the mouse fell from the wire was measured. If the mouse stayed on the metal mesh for 120 s or longer, the mouse was returned to the home cage. Mice were provided one-minute intervals between the three consecutive trials. The inverted screen test was performed by personnel blinded to the treatment group and randomized animals.

### Isolation of brain mitochondria

Mitochondria were isolated from the whole brain (minus hippocampus) using a Percoll gradient and ultracentrifugation. All steps were performed on ice. Briefly, the brain was minced with scissors in 1 mL of mitochondria isolation buffer (MIB; 225 mM mannitol, 75 mM sucrose, 6 mM K_2_HPO_4_, 1 mM EGTA, 0.1% fatty acid-free BSA, pH 7.2). This mixture was sedimented (using a microfuge) to collect the solid brain tissue. The supernatant was decanted, and the brain tissue was minced again in 1 mL of MIB with scissors. The mixture was centrifuged again, and the brain tissue was resuspended in 5 mL of fresh MIB. The brain tissue was then homogenized with a Dounce homogenizer, and the resulting homogenate was centrifuged at 1,500 × g for 5 min at 4 ºC.

Next, 15%, 23%, and 40% Percoll gradients were made using 100% Percoll in MIB. 2.3 mL of the 40% Percoll gradient was layered on the bottom of a centrifuge tube, followed by 2.3 mL of 23% Percoll (middle), then 2.3 mL of 15% Percoll (top). Five mL of supernatant (from the Dounce homogenate steps) was added to the top of the layered Percoll gradients. Using an SW28.1 Beckman rotor, tubes were centrifuged at 7,800 rpm for 13 min at 4 ºC. The mitochondrial layer was collected and washed with 8 mL of MIB and centrifuged at 8,000 × g for 10 min. Mitochondria were then washed a second time with 8 mL of sterile phosphate-buffered saline (PBS) and re-centrifuged at 8,000 × g for 10 min. Precipitated mitochondria were resuspended, gently, in 500 µL MIB.

### Oroboros respiration measurements

A total of 15 µL of isolated brain mitochondria was added to the Oroboros chamber, which contained Miro5 buffer (0.5 mM EGTA, 3 mM MgCl_2_*6H_2_O, 60 mM Lactobionic acid, 20 mM Taurine, 10 mM KH_2_PO_4_, 20 mM HEPES, 110 mM D-Sucrose, 1 g/L fatty acid free BSA). After signal equilibration (state 1), 10 mM pyruvate and 5 mM malate were added to reach state 2 respiration (the final concentration is indicated for all injections). The incubation was followed by injection of 4 mM ADP (state 3), 4 μM oligomycin (state 4o), and two injections of 0.75 μM FCCP to reach maximum respiration. The final injection consisted of 5 μM antimycin A and 1 μM rotenone. The respiratory control ratio was calculated by dividing the state 3 respiration rate by the state 4o respiration rate. Spare respiratory capacity was calculated by subtracting the FCCP respiratory rate from the state 3 rates. ATP-linked respiration was calculated by subtracting the post-oligomycin respiratory rate from the state 3 rates. All data are normalized to the protein concentration of the mitochondrial sample.

### Reverse transcription qPCR for spinal cord mRNAs

Thoracic spinal cord tissue was dissected and preserved in RNA Later. RNA isolation, cDNA synthesis, and qPCR were completed as previously described^23^. mRNA levels were measured using primers and Taqman probes from Applied Biosystems including actin (*ActB*), peroxisome proliferative activated receptor, gamma, coactivator 1 alpha (PGC1α, *Ppargc1a*), and tumor necrosis factor-α (TNFα, *Tnf*). Amplification rates of the resultant amplicons were compared to the amplification rate of actin using the ΔΔCT calculation. qPCR was completed in triplicate on each wild-type mouse, SOD1^G93A^ mouse, and SOD1^G93A^ mouse treated with oxaloacetate.

### Western blots for spinal cord proteins

Lumbar spinal cord tissue was dissected and preserved in RNA Later. Protein lysates were generated using RIPA buffer in the presence of protease and phosphatase inhibitors (Halt protease inhibitors, ThermoFisher). Western blots were completed as previously described using 4–15% Criterion TGX stain free gels (BioRad) using an antibody against nuclear factor kappa-light-chain-enhancer of activated B (NF-κB, Cell Signaling Technology #8242, used at 1:1000)^23^. This anti-NF-κB monoclonal antibody was produced by immunizing rabbits with a synthetic peptide corresponding to residues surrounding Glu498 of human NF-κB p65 protein (manufacture’s information). This monoclonal antibody recognizes mouse NF-κB but does not cross-react with other NF-κB/Rel family members (manufacture’s information) and has been cited in 412 publications (www.citeab.com). We used the BioRad Chemidoc XRS system and its Image Lab software to automatically optimize the exposure and ensure that the image signal intensity has a wide dynamic range. NF-κB signal intensity was normalized to total protein stain obtained through BioRad Stain Free Technology gels. Data are from four mice in each group, wild-type, SOD1^G93A^, and SOD1^G93A^ mice treated with oxaloacetate, in duplicate. We tested three commercially available antibodies to detect phosphorylated-NF-κB. However, we were not able to reliably analyze the level of phosphorylated-NF-κB in our hands.

### Statistics

All statistics were performed using GraphPad Prism software version 9. Significance was assessed by one-way ANOVA with Bonferroni’s multiple comparisons test for Fig. 1, unpaired *t*-test for Fig. 5, and one-way ANOVA with uncorrected Fisher’s LSD multiple comparison test for Fig. 6. The *p* and *n* values are reported in the text. The lifespans of the three groups were indicated as Kaplan–Meier survival curves using percent survival against the age of the animals. Data were statistically analyzed by Log-rank test, and survival curves between two groups were compared via a log-rank test (Mantel-Cox test).

Following data passed the Shapiro-Wiki normality test (alpha = 0.05): Fig. 1 for ages 100, 110, 120 of presymptomatic treatment group and ages 90, 100, 110, 120 of the control group; Figs. 5A, 5E, 5F for SOD1^G93A^ + OAA; 5B, 5C, 5D for SOD1^G93A^ + OAA and SOD1^G93A^; 6A for WT and SOD1^G93A^; 6B, 6C for WT, SOD1^G93A^ and SOD1^G93A^ + OAA. Following data did not pass the normality test: Fig. 1 for age 90 of presymptomatic treatment group (*p* = 0.0112); Figs. 5A, 5E, 5F for SOD1^G93A^ (*p* = 0.0303, 0.0250, 0.0250); 6A for SOD1^G93A^ + OAA (*p* = 0.0042). The small sample size may be underpowered to detect nongaussian distributions; therefore, parametric tests were used in this study.

### Data availability

The datasets generated during and analyzed during the current study are available from the corresponding author on reasonable request.
